# A Latent Variable Approach for Causal Effect Estimation Under Misclassified Treatment Assignment

**DOI:** 10.1002/sim.70528

**Published:** 2026-04-09

**Authors:** Yimeng Shang, Yu‐Han Chiu, Lan Kong

**Affiliations:** ^1^ Department of Public Health Sciences, College of Medicine Pennsylvania State University Hershey PA USA; ^2^ Department of Epidemiology Brown University School of Public Health Providence RI USA; ^3^ Center for Gerontology and Health Care Research Brown University School of Public Health Providence RI USA

**Keywords:** causal inference, EM algorithm, measurement error, misclassified treatment assignment, observational studies, validation data

## Abstract

Misclassification in treatment assignment is a common issue in causal inference with observational studies, often leading to biased estimates of causal effects if unaddressed. Several methods have been developed to handle this issue by making use of a validation dataset. This paper proposes a robust latent variable approach for causal effect estimation without the need of validation data. By employing a potential outcome modeling framework that incorporates true treatment assignment as a latent variable, we construct a likelihood function that involves three models: the outcome model, the measurement error model for misclassification, and the propensity score model for treatment assignment. To enhance the robustness against misspecification of the measurement error mechanism, we further incorporate neural networks into the estimation of the measurement error model. The simulation results show that our method performed well under various misclassification assumptions, and that using neural networks reduced the impact of misspecification of functional form for the measurement error model. We illustrate the method using a synthetic dataset derived from the Right Heart Catheterization (RHC) study. This flexible framework mitigates bias and improves the reliability of causal inference when treatment assignment is subject to misclassification and no validation data is available.

## 1 Introduction

In observational studies, measurement error in exposure, outcomes or covariates is a common issue, which could harm the reliability of the analysis results. The problem of measurement errors often arises from various sources. Ravelli et al. reported that all three traditional self‐reported dietary instruments had systematic misreporting errors [1], and similar issues with self‐reported outcomes have also been observed in other studies [2, 3]. As large administrative databases become more available for research purposes, measurement error or misclassification in treatment assignment presents a big challenge to comparative effectiveness analysis [4, 5, 6, 7, 8]. For instance, electronic medical records (EMR) usually only capture the health services and medications provided within a particular healthcare system and may not reflect the full treatment and care received by the patients. Furthermore, the inconsistent terminology that different healthcare providers use for the same condition makes it hard to capture all subjects under the same treatment. The vague or unclear descriptions in medical records add more challenges in identifying the correct treatment assignment. Misclassification may result from misdiagnosis, underdiagnosis, or a lack of clinical details in EMR or medical claims [9]. Al‐Sahab et al. provided a comprehensive overview of biases along the pathways of generating real‐world evidence for true causal relationships [8].

Traditional causal effect estimation methods, such as propensity score methods and outcome modeling all rely on accurate measurement of treatment assignment and covariates. Misclassification of treatment assignment distorts the relationship between the treatment and the outcome, undermining the validity of propensity scores and leading to incorrect adjustments for confounding variables. Consequently, the estimated treatment effects may be severely biased. Addressing treatment misclassification is therefore crucial for ensuring the accuracy and reliability of causal effect estimation in observational studies.

Measurement error problems in regression settings have been discussed intensively in the past few decades [5, 10, 11, 12]. In the context of causal inference, Babanazhad et al. demonstrated the impact of exposure misclassification on several causal estimation strategies, that is, inverse probability of treatment weighted (IPTW) estimators, propensity score adjusted estimators, and ordinary least squares estimators [13]. They developed a bias correction method to eliminate the bias using the regression calibration approach [14, 15, 16]. One major advantage of this approach is simplicity. However, this approach requires information from validation samples or other similar studies [17, 18]. When the measurement error mechanism from other studies is not transportable to the current study, this approach may lead to biased results. In addition, attention is needed both in how to develop the calibration equation and in selecting which covariates to include in the model [16]. Recently, Braun et al. proposed a two‐step pseudo‐likelihood approach to address the issue of treatment misclassification [19]. In their application, they adjusted the misclassification in both the propensity score estimation stage and outcome analysis stage based on a validation sample. Their methods worked well when propensity score stratification method was used for causal estimation, but failed to provide satisfactory results for propensity score weighting or matching method because the true treatment labeling is needed in these methods.

Since the validation sample is not always available and there is a transportability concern with the use of other similar studies, we aim to develop a causal estimation method that can address treatment misclassification without relying on validation data. Motivated by the work of Braun et al. [19], we consider a latent variable approach under the outcome regression modeling framework. By treating the true treatment assignment as a latent variable, we construct a likelihood function involving three models, that is, the propensity score (PS) model, the measurement error model, and the outcome model. The modeling of PS and outcome models is well studied in the causal inference literature. To enhance the robustness against misspecification of the measurement error mechanism, we further incorporate neural networks into the estimation of measurement error model.

The remainder of this paper is organized as follows. In Section 2, we lay out the general model framework of latent variable approach and present the estimation procedure via an Expectation–Maximization (EM) algorithm. Through comprehensive simulation studies in Section 3, we first assess the performance of the proposed methods under a variety of scenarios, in comparison with Naïve methods which ignore the treatment misclassification. Then we evaluate the robustness of our methods to the misspecification of the functional form of measurement error model. In Section 4 we apply the proposed methods to a synthetic dataset based on an observational study that investigated the causal effect of right heart catheterization on the hospital length of stay. We provide conclusions and discussions in Section 5.

## 2 Methods

In this section, we first describe the causal effect identification using a g‐computation method. Then we describe the general framework of our latent variable approach that consists of three models: outcome model, measurement error model, and propensity score (PS) model for treatment assignment. Next we discuss how our proposed method may adopt the flexible modeling of misclassification, and incorporate potential validation data when available. Lastly, we present an EM algorithm for model estimation.

### 2.1 Causal Effect Identification

Suppose there is a random sample of n individuals, let $T_{i}$ denote correctly labeled binary treatment for the ith subject, which is a latent variable that can not be observed. Let $T_{i}^{obs}$ represent observed treatment with potential misclassification error, which is used as the surrogate of $T_{i}$. The full set of confounders for the ith subject is denoted by $X_{i}={\left(X_{i1},X_{i2},\ldots ,X_{ip}\right)}^{T}$, where p is the dimension of the covariates vector. Let $Y_{i}$ denote the outcome for each subject and assume that it follows a distribution from an exponential family.

To find the causal relationships, we make the classic assumptions for the counterfactual framework as discussed by Rubin [20], including the exchangeability, stable unit treatment value assumption (SUTVA), consistency, and positivity. In addition, we assume non‐differential misclassification regarding the measurement error [21], that is,

A.1
$f(Y|T^{obs},T=t,X)=f(Y|T=t,X)$. The distribution of Y is conditionally independent of the observed treatment assignment.

To estimate the marginal causal effect, we can employ g‐computation, also known as parametric g‐formula or (g‐) standardization [22, 23]. The counterfactual outcome $Y_{i}(1)$ and $Y_{i}(0)$ for subject i under treatment $T_{i}=1$ and 0 can be identified by consistency assumption [24]:

$$\begin{array}{ll} E\left(Y_{i}(a)|X_{i}\right) & =E\left(Y_{i}(a)|T_{i}=a,X_{i}\right)\\ & =E\left(Y_{i}|T_{i}=a,X_{i}\right),\text{where a}=0,1. \end{array}$$

Thus,

$$\begin{array}{l} E\left(Y_{i}(a)\right)=E\left[E\left(Y_{i}(a)|X_{i}\right)\right]=E\left[E\left(Y_{i}|T_{i}=a,X_{i}\right)\right] \end{array}$$

Average treatment effect (ATE) is defined as:

$$\begin{array}{l} ATE=E\left(Y_{i}(1)\right)-E\left(Y_{i}(0)\right) \end{array}$$

### 2.2 Model Description

#### 2.2.1 Outcome Model

We consider a generalized linear model as follows:

$$\begin{array}{ll} E\left(Y_{i}|T_{i}^{obs},T_{i},X_{1i}\right) & \overset{\text{A.1}}{=}E\left(Y_{i}|T_{i},X_{1i};\psi ,\gamma_{0},\gamma \right)\\ & =g^{-1}\left(\gamma_{0}+\psi T_{i}+X_{1i}^{T}\gamma \right),\;(M1) \end{array}$$

where g(.) is the link function, $\gamma_{0}$ is the intercept, ψ is the coefficient for true treatment assignment and γ is the coefficient vector associated with a vector of covariates, $X_{1i}$, which can be a subset of $X_{i}$.

With the outcome model estimated, the counterfactual outcomes $Y_{i}(1)$ and $Y_{i}(0)$ for subject i under treatment $T_{i}=1$ and 0 can be predicted by fitting the outcome model M1, that is,

$$\begin{array}{l} {\hat{Y}}_{i}(t)|X_{1i}=E\left[Y_{i}|T_{i}=t,X_{1i};\hat{\psi },\hat{\gamma }\right]=g^{-1}\left({\hat{\gamma }}_{0}+\hat{\psi }T_{i}+X_{1i}^{T}\hat{\gamma }\right) \end{array}$$

Thus, the marginal average treatment effect can be estimated by taking the average of the difference between ${\hat{Y}}_{i}(1)$ and ${\hat{Y}}_{i}(0)$ across all subjects:

$$\begin{array}{lll} \hat{\text{ATE}} & =\hat{Y}(1)-\hat{Y}(0),\;\text{where}\;\hat{Y}(t)\; & =\frac{1}{n}\overset{n}{\underset{i=1}{\sum }}{\hat{Y}}_{i}(t) \end{array}$$

For a continuous outcome, the ATE is represented by the parameter ψ when the identity link function is used. For a binary outcome under the logit link function, a marginal causal odds ratio (OR) can be calculated by g‐computation [25] as follows:

$$\begin{array}{ll} \hat{\text{OR}} & =\frac{\hat{P}(Y(1)=1)/\hat{P}(Y(1)=0)}{\hat{P}(Y(0)=1)/\hat{P}(Y(0)=0)},\\ \;\text{where}\;\hat{P}(Y(t)=1) & =\frac{1}{n}\overset{n}{\underset{i=1}{\sum }}P\left(Y_{i}=1|T_{i}=t,X_{1i};\hat{\psi },\hat{\gamma }\right)\\ & =\frac{1}{n}\overset{n}{\underset{i=1}{\sum }}E\left[Y_{i}|T_{i}=t,X_{1i};\hat{\psi },\hat{\gamma }\right]. \end{array}$$

#### 2.2.2 Measurement Error Model for Misclassification

The measurement error model characterizes the mechanism of measurement error, or classification error in this case. Given the true treatment assignment $T_{i}$ and covariate vector $X_{2i}$, the observed $T_{i}^{obs}$ can be described by a logistic regression:

$$\begin{array}{ll} E\left(T_{i}^{obs}|T_{i},X_{2i};\alpha ,\eta \right) & =exp\left(\alpha_{0}+\alpha_{1}T_{i}+X_{2i}^{T}\eta \right)/\\ & \;\left\{1+exp\left(\alpha_{0}+\alpha_{1}T_{i}+X_{2i}^{T}\eta \right)\right\},\;(M2) \end{array}$$

where $\alpha =(\alpha_{0},\alpha_{1})$ are the coefficients for intercept and true treatment assignment and η is the coefficient vector associated with a vector of covariates, $X_{2i}$, which can be a subset of $X_{i}$.

The measurement error model serves as a nuisance model, which is sometimes challenging to model due to the lack of clear prior knowledge about the error mechanism. When the measurement error model is misspecified, the causal effect estimation is biased and has a large variance. We propose using machine learning algorithms for measurement error modeling to enhance flexibility and avoid dependence on precise model specifications. For example, a neural network (NN) model can be applied to predict $P[T_{i}^{obs}=1|T_{i},X_{2i}]=h(X_{2i})$, where h(.) is a non‐parametric function of $X_{2i}$. In addition, if the true treatment assignment is available for a small validation cohort, the validation sample can be used to estimate the parameters in the measurement error model when the main data have the same measurement error mechanism.

#### 2.2.3 Propensity Score Model for Treatment Assignment

The propensity score model is a key component for causal inference. When there is misclassification in treatment assignment, the estimated propensity score may fail to balance covariates between treatment groups. We assume a logistic regression model for the latent true treatment assignment:

$$\begin{array}{ll} & P\left(T_{i}=1|X_{3i};\beta_{0},\beta \right)\\ & \;=exp\left(\beta_{0}+X_{3i}^{T}\beta \right)/\left\{1+exp\left(\beta_{0}+X_{3i}^{T}\beta \right)\right\},\;(M3) \end{array}$$

where $\beta_{0}$ is the intercept, β is the coefficient vector associated with a vector of covariates, $X_{3i}$, which can be a subset of $X_{i}$.

### 2.3 Joint Likelihood Function

Let θ represent all the parameters in the three models described above. The likelihood function for the observed data $O_{i}=(Y_{i},X_{i},T_{i}^{obs})$ can be written as:

$$\begin{array}{ll} & L_{o}\left(\theta |Y_{i},T_{i}^{obs},X_{i}\right)=\overset{n}{\underset{i=1}{\prod }}f\left(Y_{i},T_{i}^{obs},X_{i}\right)\\ & \;=\overset{n}{\underset{i=1}{\prod }}\underset{t\in 0,1}{\sum }f\left(Y_{i},T_{i}=t,T_{i}^{obs},X_{i}\right)\\ & \;=\overset{n}{\underset{i=1}{\prod }}\underset{t\in 0,1}{\sum }f\left(Y_{i}|T_{i}^{obs},T_{i}=t,X_{i}\right)\\ & \;\cdot P\left(T_{i}^{obs}|T_{i}=t,X_{i}\right)\cdot P\left(T_{i}=t|X_{i}\right)\cdot f\left(X_{i}\right)\\ & \;\overset{\text{A.1}}{=}\overset{n}{\underset{i=1}{\prod }}\underset{t\in 0,1}{\sum }\;\underset{M1}{\underbrace{f\left(Y_{i}|T_{i}=t,X_{i};\psi ,\gamma \right)}}\\ & \;\cdot \underset{M2}{\underbrace{P\left(T_{i}^{obs}|T_{i}=t,X_{i};\alpha ,\eta \right)}}\cdot \underset{M3}{\underbrace{P\left(T_{i}=t|X_{i};\beta \right)}}\cdot f\left(X_{i}\right)\\ & \;\text{where}\;\theta ={\left(\psi ,\gamma^{T},\alpha^{T},\eta^{T},\beta^{T}\right)}^{T} \end{array}$$

### 2.4 Estimation Procedure

Because directly optimizing the observed likelihood is challenging, we employ the EM algorithm [26] to find the maximum likelihood estimate of θ. The complete‐data likelihood function based on the observed data $O_{i}$ and unobserved $T_{i}$ can be written as:

$$\begin{array}{ll} & L_{c}\left(\theta |Y_{i},T_{i},T_{i}^{obs},X_{i}\right)=\overset{n}{\underset{i=1}{\prod }}P\left(Y_{i},T_{i},T_{i}^{obs},X_{i}\right)\\ & \overset{\text{A.1}}{=}\overset{n}{\underset{i=1}{\prod }}\underset{t\in 0,1}{\prod }\left\{f\left(Y_{i}|T_{i}=t,X_{i};\psi ,\gamma \right)\cdot P\left(T_{i}^{obs}|T_{i}=t,X_{i};\alpha ,\eta \right)\right.\\ & \;{\left.\cdot P\left(T_{i}=t|X_{i};\beta \right)\cdot f\left(X_{i}\right)\right\}}^{I(T_{i}=t)}\\ & \propto \overset{n}{\underset{i=1}{\prod }}\underset{t\in 0,1}{\prod }\left\{f\left(Y_{i}|T_{i}=t,X_{i};\psi ,\gamma \right)\cdot P\left(T_{i}^{obs}|T_{i}=t,X_{i};\alpha ,\eta \right)\right.\\ & \;{\left.\cdot P\left(T_{i}=t|X_{i};\beta \right)\right\}}^{I(T_{i}=t)} \end{array}$$

Thus, the complete‐data log‐likelihood function is given by:

$$\begin{array}{ll} & l_{c}\left(\theta |Y_{i},T_{i},T_{i}^{obs},X_{i}\right)\\ & \;\propto \overset{n}{\underset{i=1}{\sum }}\underset{t\in 0,1}{\sum }I(T_{i}=t)\log f\left(Y_{i}|T_{i}=t,X_{i};\psi ,\gamma \right)+\\ & \;\overset{n}{\underset{i=1}{\sum }}\underset{t\in 0,1}{\sum }I(T_{i}=t)\log P\left(T_{i}^{obs}|T_{i}=t,X_{i};\alpha ,\eta \right)+\\ & \;\overset{n}{\underset{i=1}{\sum }}\underset{t\in 0,1}{\sum }I(T_{i}=t)\log P\left(T_{i}=t|X_{i};\beta \right) \end{array}$$

The E‐step involves estimating the expectation of $T_{i}$ given observed data $O_{i}$ and current estimate of θ at iteration r:

ξi^(r)≡ETi|Yi,Tiobs,Xi;θ^(r) =PTi=1|Yi,Tiobs,Xi;θ^(r) =A.1 fYi|Ti=1,Xi;ψ^(r),γ^(r)·PTiobs|Ti=1,Xi;α^(r),η^(r)·PTi=1|Xi;β^(r)∑t∈0,1fYi|Ti=t,Xi;ψ^(r),γ^(r)·PTiobs|Ti=t,Xi;α^(r),η^(r)·PTi=t|Xi;β^(r) 

In the (r+1)th iteration of M‐step, we obtain the updated estimate ${\hat{\theta }}^{\left(r+1\right)}$ by maximizing the expected log‐likelihood function given the current estimate, ${\hat{\theta }}^{\left(r\right)}$, as follows:

$$\begin{array}{ll} Q\left(\theta |{\hat{\theta }}^{\left(r\right)}\right) & \equiv E\left\{l_{c}\left(\theta |Y_{i},T_{i}^{obs},X_{i},{\hat{\theta }}^{\left(r\right)}\right)\right\}\\ & \propto E\left[\overset{n}{\underset{i=1}{\sum }}\underset{t\in 0,1}{\sum }I(T_{i}=t)\log\left\{f\left(Y_{i}|T_{i}=t,X_{i};\psi ,\gamma \right)\right.\right.\\ & \;\left.\left.\cdot P\left(T_{i}^{obs}|T_{i}=t,X_{i};\alpha ,\eta \right)\cdot P\left(T_{i}=t|X_{i};\beta \right)\right\}\right]\\ & =\overset{n}{\underset{i=1}{\sum }}\underset{t\in 0,1}{\sum }P\left(T_{i}=t|Y_{i},T_{i}^{obs},X_{i};{\hat{\theta }}^{\left(r\right)}\right)\\ & \;\cdot \left\{\log f\left(Y_{i}|T_{i}=t,X_{i};\psi ,\gamma \right)+\right.\\ & \;\log P\left(T_{i}^{obs}|T_{i}=t,X_{i};\alpha ,\eta \right)+\\ & \;\left.\log P\left(T_{i}=t|X_{i};\beta \right)\right\} \end{array}$$

The expected log‐likelihood comprises the three models (M1,M2,M3) we defined in Section 2.2. To maximize it, we optimize each component individually. Consequently, the optimization reduces to maximizing the likelihood function of each weighted model, with weights defined as the ${\hat{\xi_{i}}}^{\left(r\right)}$ in the previous E‐step.

The choices of initial values are critical for optimization. Specifically, we fit the outcome model M1 and propensity score model M3 with the latent true treatment assignment $T_{i}$ replaced by the observed treatment assignment $T_{i}^{obs}$ and then use the parameter estimates as the initial values. For the parameters in the measurement error model M2, researchers may select reasonable estimates of the sensitivity and specificity of surrogate $T_{i}^{obs}$ based on prior knowledge and then set the initial values of parameters as $\alpha_{0}=logit(1-\text{specificity})$ and $\alpha_{1}=logit(\text{sensitivity})-\alpha_{0}$. Since the error mechanism is likely unknown, we may set initial β to be 0 for simplicity. We repeat the E‐step and M‐step until the convergence criteria are met or the maximum number of iterations is reached.

## 3 Simulation

A series of simulation studies are conducted to evaluate various aspects of the proposed method. We start with a simple case where the misclassification is conditionally independent of covariates, that is, $T^{obs}$ and X are independent given true treatment assignment T. Under this setting, we also investigate how the degree of measurement error influences the performance by varying the sensitivity and specificity of $T^{obs}$ in measuring true treatment assignment. Then we move to the case when $T^{obs}$ depends on both X and T. In this more general case, we examine the performance of our proposed methods for estimating marginal causal effect under different types of outcomes, including continuous and binary outcomes. In addition, we incorporate the neural network approach in the estimation of the measurement error model and investigate the robustness of our method under both correctly specified and misspecified measurement error model. Lastly, we consider the case when the true treatment assignment is known for a validation sample. We compare our proposed methods, which do not rely on the validation sample, to the approach where the validation sample is used to estimate the measurement error model as benchmark.

### 3.1 Simulation Set‐Up

We consider five covariates, $X_{1}$ to $X_{5}$, in the simulation study, all of which are confounders. Three continuous variables, $X_{1}$ to $X_{3}$, are generated from a multivariate Gaussian distribution with mean zero and covariance matrix ∑=AR1(0.3), a first‐order autoregressive correlation structure with variance equal to 1. Two binary variables, $X_{4}$ and $X_{5}$, are generated from a Bernoulli distribution, Bern(0.5).

Next, we use the logistic regression models as described in Section 2.1 to simulate the true treatment assignment (T) and misclassified assignment ($T^{obs}$). The continuous and binary outcome data (Y) are generated via linear regression and logistic regression models, respectively. The detailed model settings are provided in the following section.

For each simulation scenario, we conducted 1000 Monte Carlo simulations with a varying sample size. We compare the performances of our proposed methods with two methods: the Naïve method that ignores the misclassification in treatment assignment; and the Oracle method, which uses the true treatment assignment in the outcome modeling and serves as the best‐case standard. The bias and root‐mean‐square error (RMSE) are reported for the estimates of the average treatment effect (ATE). The estimating procedure was implemented in R 4.2.1. Source code for simulations and algorithm instructions are publicly available on GitHub (https://github.com/ys3298/MissclassifiedT_latentVar.git).

### 3.2 Simulation Results

#### 3.2.1 Scenario 1: Misclassification is Independent of Covariates

As shown in the directed acyclic graph (DAG) in Table 1, Scenario 1 assumes conditional independence of $T^{obs}$ and X given T. X serves as the confounders and the covariates imbalance in treatment groups and the association between X and outcome Y are strong. When the measurement error model is independent of X, the value of α can be uniquely mapped to the sensitivity and specificity of $T^{obs}$ given T. We choose the parameters α to have sensitivity and specificity at 80%. The variance of outcome is set to $\sigma^{2}=1$. The true ATE is 2.0 in this setting.

**TABLE 1:** Simulation setting for Scenario 1.

DAG	Description and parameters
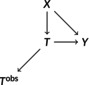	$logit(E\left[T^{obs}|T;\alpha \right])=-1.4+2.8T$
	($T^{\text{obs}}$ does not depend on X)
	$logit(E\left[T|X;\beta \right])=-2.0X_{1}+3.0X_{2}+1.0X_{3}+0.5X_{4}+0.5X_{5}$
	$E\left[Y|T,X;\psi ,\gamma \right]=1+2.0T-0.8X_{1}+0.8X_{2}-0.8X_{3}+0.8X_{4}-0.8X_{5}$, $\sigma^{2}=1$

**FIGURE 1:**
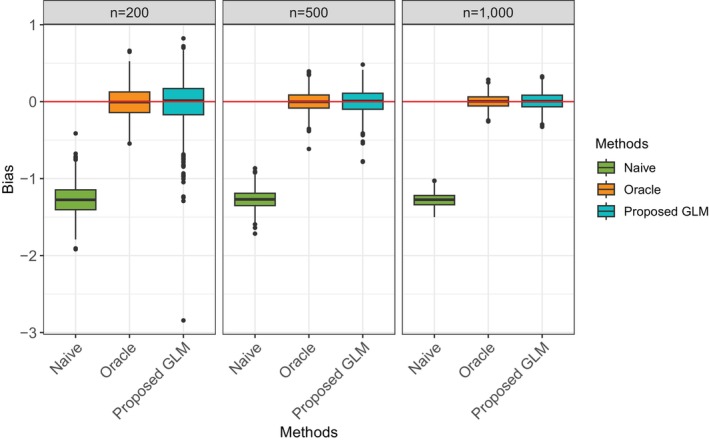
Boxplot of ATE estimation bias from 1000 simulation runs for Scenario 1.

Table 2 presented the bias and RMSE of each method under scenario 1 when sample size n=200, n=500, and n=1000. When applying the proposed approach with generalized linear model (GLM) as the measurement error model, we assume that the true underlying measurement error mechanism is unknown and include both T and X into the model, although X is not involved in generating the $T^{obs}$. From Figure 1, we can observe that Naïve has a large estimation bias due to ignoring the misclassified treatment assignment. The proposed method with GLM as M2 significantly reduces the bias compared with the Naïve method. Compared with the Oracle approach, it has a slightly higher variance. With the increase in sample size, the variance is reduced.

**TABLE 2:** ATE estimation bias (RMSE) under for Scenario 1.

	Naïve	Oracle	Proposed GLM
n = 200	−1.274(1.290)	−0.004(0.206)	−0.014(0.305)
n = 500	−1.271(1.276)	0.000 (0.132)	0.006 (0.159)
n = 1000	−1.275(1.278)	0.004 (0.089)	0.007 (0.110)

We are also interested in the effect of the degree of measurement error on the performance of the proposed method. To examine how the magnitude of measurement error impacts the performance of the proposed method, we varied the sensitivity and specificity of the measurement error model with a sample size of 1000.

**FIGURE 2:**
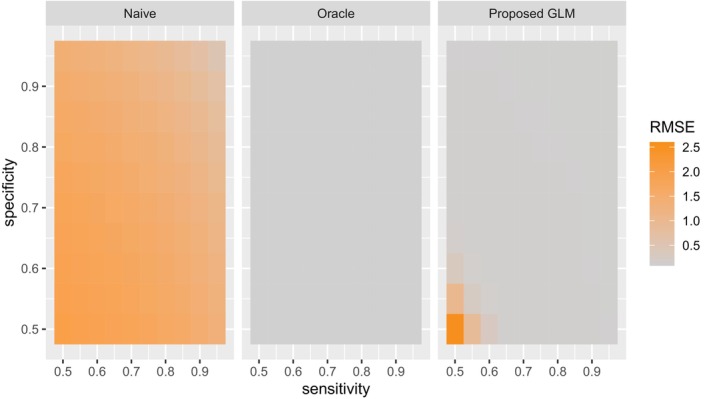
Heatmap of RMSE of ATE estimates with varying sensitivity and specificity.

Figure 2 presented the change of RMSE with the change of sensitivity and specificity for each method. As noticed, the proposed methods perform well with small RMSE for all sets of sensitivity and specificity except when both sensitivity and specificity are closed to 0.5. However, the RMSE of the Naïve method increases dramatically with the decrease of sensitivity and specificity. This is expected since $T^{obs}$ distorts more from T when the sensitivity and specificity decrease.

#### 3.2.2 Scenario 2: Misclassification Depends on Covariates in a Linear Form

**FIGURE 3:**
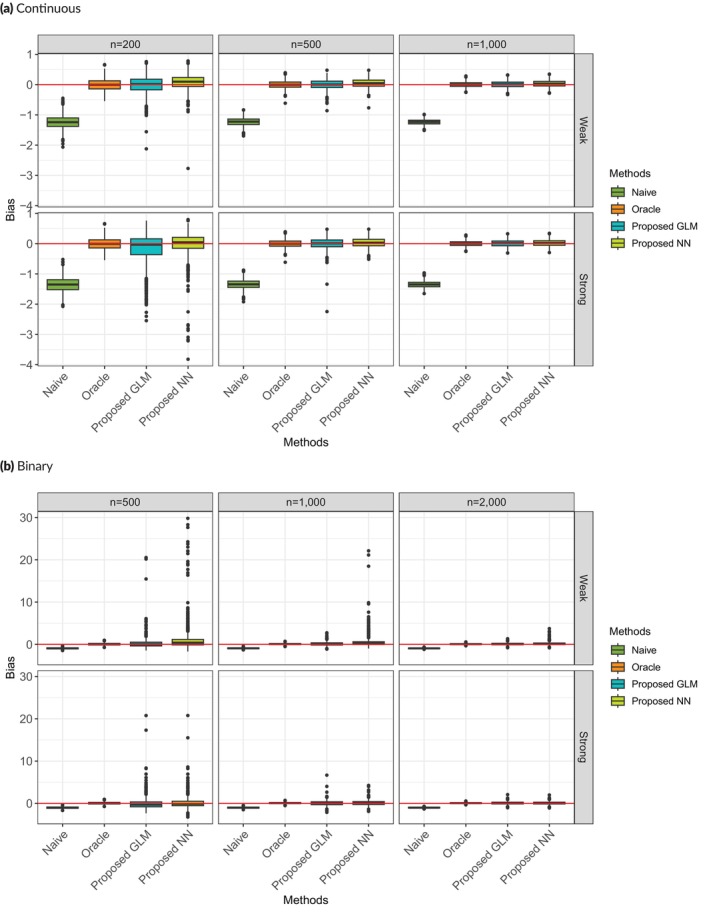
Boxplots of ATE estimation bias for Scenario 2.

For Scenario 2, the structure is more complicated in the sense that $T^{obs}$ depends not only on true classification T but also on other covariates X (Table 3) either strongly or weakly. In this scenario, we consider both continuous and binary outcomes and use GLM and NN as the measurement error model, respectively, in the estimation. We can examine how robust the NN method is when GLM is the true measurement error mode. Table 4 and Figure 3 present the performance of different methods with sample sizes 200, 500, and 1000 for continuous outcomes and 500, 1000, and 2000 for binary outcomes. Although the measurement error mechanism is more complicated than that in scenario 1, the proposed GLM method still performs well for the continuous outcome as indicated by small bias and variance comparable to the Oracle method. In the case of strong dependence on X, a larger sample size would improve the performance of GLM. In comparison, the proposed NN method also yields satisfactory results. For binary outcome, the true conditional effect is 2, and the marginal effect is 1.47. The proposed GLM method performs much better than the Naïve method, but the bias and variance are larger compared to Oracle method. However, the performance improves with larger sample size and stronger dependence. This is also the case for the NN method, and a much larger sample size is required for the NN method to achieve reasonable performance.

**TABLE 3:** Simulation setting for Scenario 2.

DAG	Description and parameters
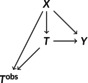	$logit(E\left[T^{obs}|T,X;\alpha ,\eta \right])=-1.4+2.8T-0.4X_{1}+0.4X_{3}-0.4X_{5}$
	($T^{\text{obs}}$ depends on T and **weakly** on X)
	$logit(E\left[T^{obs}|T,X;\alpha ,\eta \right])=-1.4+2.8T-2.0X_{1}+2.0X_{3}-2.0X_{5}$
	($T^{\text{obs}}$ depends on T and **strongly** on X)
	$logit(E\left[T|X;\beta \right])=-2.0X_{1}+3.0X_{2}+1.0X_{3}+0.5X_{4}+0.5X_{5}$
	$E\left[Y|T,X;\psi ,\gamma \right]=1+2.0T-0.8X_{1}+0.8X_{2}-0.8X_{3}+0.8X_{4}-0.8X_{5}$, $\sigma^{2}=1$
	$logit(E[Y|T,X;\psi ,\gamma ])=-1.0+2.0T-0.8X_{1}+0.8X_{2}-0.8X_{3}+0.8X_{4}-0.8X_{5}$

**TABLE 4:** ATE estimation bias (RMSE) of different methods under different sample sizes for Scenario 2.

(a) Continuous outcome					
		Naïve	Oracle	Proposed GLM	Proposed NN
Weak dependence	*n* = 200	−1.241(1.260)	−0.004(0.206)	−0.013(0.294)	0.079 (0.267)
	*n* = 500	−1.230(1.237)	0.000 (0.132)	0.007 (0.160)	0.048 (0.154)
	*n* = 1000	−1.237(1.240)	0.004 (0.089)	0.007 (0.111)	0.030 (0.109)
Strong dependence	*n* = 200	−1.352(1.375)	−0.004(0.206)	−0.174(0.544)	−0.026(0.445)
	*n* = 500	−1.344(1.353)	0.000 (0.132)	0.004 (0.183)	0.033 (0.158)
	*n* = 1000	−1.348(1.352)	0.004 (0.089)	0.008 (0.113)	0.020 (0.111)

(b) Binary outcome					
		Naïve	Oracle	Proposed GLM	Proposed NN
Weak dependence	*n* = 500	−0.930(0.947)	0.057 (0.278)	0.206 (1.334)	1.148 (3.327)
	*n* = 1000	−0.925(0.933)	0.077 (0.203)	0.123 (0.441)	0.534 (1.597)
	*n* = 2000	−0.936(0.940)	0.062 (0.147)	0.081 (0.286)	0.155 (0.418)
Strong Dependence	*n* = 500	−1.012(1.035)	0.057 (0.278)	−0.058(1.383)	0.129 (1.378)
	*n* = 1000	−1.008(1.020)	0.077 (0.203)	0.048 (0.598)	0.100 (0.566)
	*n* = 2000	−1.009(1.015)	0.062 (0.147)	0.092 (0.345)	0.090 (0.347)

#### 3.2.3 Scenario 3: Misclassication Depends on Covariates in a Non‐Linear Form

In this scenario, we consider a more complex measurement error model where the misclassification depends on covariates in a non‐linear form and there is an interaction between covariates (Table 5). To investigate the impact of a misspecified measurement error model on the ATE estimation, we fit a misspecified measurement error model, where only the linear term of covariates are included in the GLM model. We compare the performance of this GLM approach with that based on the neural network approach.

**TABLE 5:** Simulation setting for Scenario 3.

DAG	Description and parameters
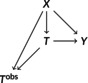	$logit(E\left[T^{obs}|T,X;\alpha ,\eta \right])=-1.4+2.8T-2.0e^{X_{1}/2}+2.0{\left(\frac{X_{1}X_{3}}{25}+0.6\right)}^{3}-2.0X_{5}$
	$logit(E\left[T|X;\beta \right])=-2.0X_{1}+3.0X_{2}+1.0X_{3}+0.5X_{4}+0.5X_{5}$
	$E\left[Y|T,X;\psi ,\gamma \right]=1+2.0T-0.8X_{1}+0.8X_{2}-0.8X_{3}+0.8X_{4}-0.8X_{5}$, $\sigma^{2}=1$

We chose a one‐hidden‐layer neural network in which the activation function is ReLU [27] for the hidden layer and the sigmoid function for the output layer for measurement error modeling. Specifically, we employ 3‐fold cross‐validation (CV) to perform a grid search for tuning parameters, selecting the number of neurons in the hidden layer (ranging from 1 to 3) and the learning rate decay (ranging from 0 to 0.1).

**FIGURE 4:**
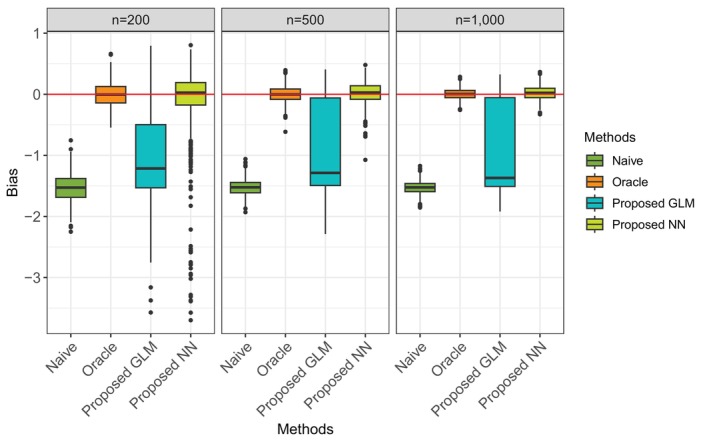
Boxplot of ATE estimation bias for Scenario 3.

Table 6 and Figure 4 illustrate the comparison of various approaches when the measurement error model is misspecified in the GLM approach. When the measurement model is misspecified, the GLM method exhibits a very large bias and variance as expected. The neural network method has a very small bias for all sample sizes. However, the variance for the neural network method is relatively large when the sample size is very small (*n* = 200). With the increase in sample sizes, the variance is reduced and the NN method becomes stable, which shows robustness to model misspecification in terms of functional form.

**TABLE 6:** ATE estimation bias (RMSE) for Scenario 3.

Sample size	Naïve	Oracle	Proposed GLM	Proposed NN
*n* = 200	−1.529(1.545)	−0.004(0.206)	−1.021(1.241)	−0.071(0.534)
*n* = 500	−1.528(1.534)	0.000 (0.132)	−0.915(1.171)	0.023 (0.170)
*n* = 1000	−1.527(1.531)	0.004 (0.089)	−1.001(1.220)	0.019 (0.113)

#### 3.2.4 Scenario 4: Validation Sample is Available

As we described in the Methods section (Section 2), the proposed framework can incorporate the data from the validation sample to better estimate the measurement error model. We conduct simulation studies when the validation cohort is a random sample of the full cohort and shares the same measurement error mechanism as the full cohort.

We use the settings in Scenario 2 to generate data from the measurement error model with a strong dependence on X, continuous outcome model, and propensity score model. A validation cohort is randomly selected from the full cohort so that the measurement error mechanism is consistent in the validation subcohort and full cohort. The full cohort size is set to be n=1000, with 20% or 40% of the full cohort serving as the validation cohort where a true treatment assignment is known. We apply three methods for ATE estimation under the proposed framework. The first two rely on the GLM approach and neural network approach to address misclassification issues without using the validation data. The third one, namely the VD method, leverages true treatment assignment information from the validation cohort to estimate misclassification errors. Correctly specified measurement error models are used in the GLM and VD methods.

**FIGURE 5:**
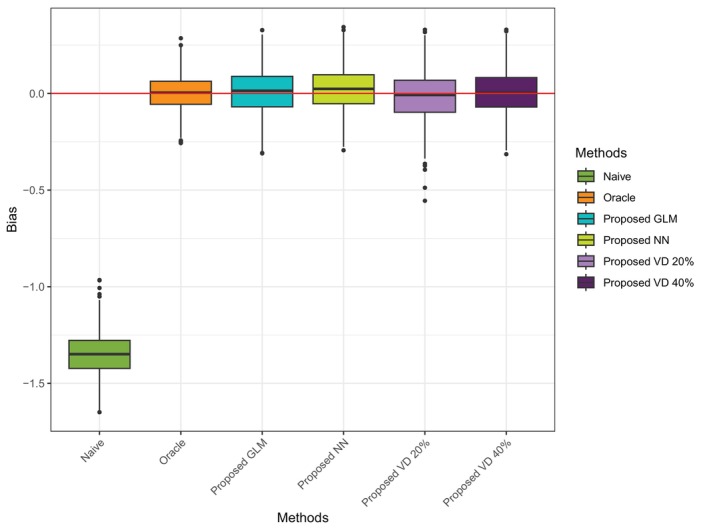
Boxplot of ATE estimation bias when 20% and 40% available as validation cohort.

As shown in Table 7 and Figure 5, all the proposed methods perform much better than the Naïve method. Without relying on the true classification in the validation cohort, the proposed GLM and NN methods perform as well as the VD method when only a small proportion of the validation cohort (20%) relative to the full cohort is available. With a larger validation cohort (40%), the performance of the VD method improves significantly, as indicated by the much smaller bias and reduced variance.

**TABLE 7:** ATE estimation bias (RMSE) of different methods when validation cohort is available.

Naïve	Oracle	Proposed GLM	Proposed NN	Proposed VD 20%	Proposed VD 40%
−1.348(1.352)	0.004 (0.089)	0.008 (0.113)	0.020 (0.111)	−0.015(0.123)	0.001 (0.113)

*Note:* The sample size for the full cohort is n=1000, with 200 for the Proposed VD 20%, and 400 for the Proposed VD 40% validation data.

## 4 Data Application

We demonstrated our proposed methods using a synthetic dataset derived from the Right Heart Catheterization (RHC) study by Connors et al. (1996) [28]. This observational study was designed to evaluate the impact of the utilization of right heart catheterization within the initial 24‐h period of care in the intensive care unit (ICU) on the hospital length of stay. The study cohort had 5734 patients in total, consisting of 2183 RHC users and 3551 non‐users. A panel of specialists in critical care identified a list of covariates for the study, including age, sex, race, years of education, income, medical insurance type, disease category, admission diagnosis, and other measurements.

We assumed the original RHC study had the correct classification of RHC use and generated a synthetic dataset to induce the misclassification of RHC use. Firstly, we kept all continuous covariates and those binary covariates with a prevalence greater than 20%, resulting in a total of 33 covariates. Next, the misclassified treatment assignments, $T_{i}^{obs}$, were generated using a measurement error model in the form of logistic regression:

(1) $$\begin{array}{l} \log\frac{P\left(T_{i}^{obs}=1|T_{i},X_{i}^{T}\right)}{1-P\left(T_{i}^{obs}=1|T_{i},X_{i}^{T}\right)}=\alpha_{0}+\alpha_{1}T_{i}+X_{i}^{T}\eta . \end{array}$$

where *T* is the true classification and *X* are the covariates that potentially affect the misclassification of RHC use. We select $\alpha_{0}=-1.1$ and $\alpha_{1}=-2.8$. For simplicity, we included age, education, Disease Activity Score (DAS) index, and Acute Physiology and Chronic Health Evaluation (APACHE) score in the model and set the coefficients η=(0.5,−0.5,0.7,−0.7). Under this setting, the sensitivity of using $T_{i}^{obs}$ to identify RHC user was 77.6% and the specificity was 68.7%.

We estimated the causal effect of RHC use with the proposed latent variable approach. Specifically, we fit the outcome model using linear regression, and the propensity score model using logistics regression, adjusting all 33 covariates linearly. For the measurement error model, we consider two estimation methods, parametric GLM (logistic regression) and neural network (NN) as described in the simulation study. The model includes four covariates in the true model and additional four covariates (Glasgow Coma Score, blood pressure, white blood count, and heart rate). For comparison, we also estimated the causal effects using the Naïve and Oracle methods. The estimation results are shown in Table 8. Without treatment misclassification, the Oracle estimate indicted that RHC use reduced the hospital length of stay by 3.30 days. This result is similar to those reported in the literature [29, 30]. The Naïve method significantly underestimated the causal effect. Both of our proposed methods (GLM and NN) reduced the estimation bias compared to the Naïve method, with the proposed NN method yielding the smallest bias. These results are consistent with our simulation findings. The proposed latent variable framework with the flexible measurement error model such as NN demonstrated a robust performance.

**TABLE 8:** Comparison of methods for causal effect estimation.

Method	Estimation	Bias
Oracle	3.30	—
Naïve	1.63	−1.67
GLM	2.40	−0.90
NN	3.18	−0.12

## 5 Conclusions and Discussions

Misclassification of treatment assignments is one of the many challenges associated with estimating causal effects in observational studies. The Naïve method, which treats misclassified treatment assignments as true treatment assignments, often results in bias by blending the two treatment groups. To address this issue, we propose a latent variable approach that handles the true treatment assignments as a latent variable. Based on the model assumptions for outcomes, measurement errors, and propensity scores of treatment assignments, we construct a complete‐data likelihood function and present an EM algorithm for model estimation. It is worth noting that the expected log‐likelihood function can be decomposed into the weighted likelihood functions of three models: the outcome model, the measurement error model, and the propensity score model. This decomposition enhances its interpretability and facilitates modeling by incorporating prior information for each model. The advantage of our proposed method is that validation data are not required to model measurement errors. Representative validation data is valuable since it allows us to build a model on the validation set and then apply it to the main dataset with misclassification. However, such validation data is not always available, and even when it is, it is often of lower quality than the main dataset, lacking a similar measurement error mechanism. We innovatively proposed using neural networks to model the measurement error, enabling a better capture of the relationships among variables that influence the misclassification.

As demonstrated in the simulation studies, the proposed method with a correctly specified measurement error model yielded unbiased estimates of average treatment effect. Moreover, the utilization of neural networks still produced approximately unbiased estimates when the traditional logistic regression failed to specify the measurement error model correctly. The framework can easily accommodate other probabilistic machine learning methods, such as gradient boosting. The choice of method should consider factors such as sample size, model complexity, and prior knowledge of the measurement error structure. Generalized linear models are suitable for approximately linear relationships or limited data, whereas more flexible machine learning approaches are preferable for complex, nonlinear mechanisms when sufficient data are available.

Main/validation analyses have been widely applied to adjust for measurement error in exposures. When external validation data are used to address misclassification, a key challenge is the transportability assumption, which is often difficult to assess empirically. When transportability can be reasonably justified, or when representative internal validation data are available, researchers may proceed with a main/validation analysis, such as classical regression calibration approaches [15]. More recent work has extended these frameworks to settings with internal or external validation data, including longitudinal designs and complex outcomes [31]. In such settings, calibration‐based approaches are attractive because they offer transparent bias correction with relatively weak modeling assumptions. In contrast, latent‐variable models may be preferable when validation data are sparse, non‐transportable, or unavailable, as they allow joint modeling of measurement error and outcomes using main observed data alone. These approaches are complementary rather than competing: the choice between them depends on study design, data availability, and the plausibility of underlying assumptions. Our proposed method can incorporate validation data to inform the measurement error model when available, or operate without validation data using flexible modeling. In both cases, the method remains useful for improving robustness and may offer efficiency gains, particularly when the validation dataset has a limited sample size.

The validity of our proposed method relies on whether the model assumptions are correct. In addition to the standard assumptions for propensity score based causal inference methods and the outcome model based g‐computation methods, we make one more assumption that the distribution of outcome is independent of the observed treatment assignment conditional on the true treatment assignment and confounders. This implies that after accounting for both the true treatment assignment and confounders, the misclassified treatment assignment does not provide any additional information about the outcome. This assumption is commonly made in the measurement error literature [32], and allows for the straightforward identification of the causal effect of treatment. The proposed neural network method is robust to the misspecification of functional form for the measurement error model, but it still requires access to all relevant covariates that influence misclassification. To relax the assumption of correct model specification of the propensity score and outcome models, some methods have been developed, such as the double machine learning approach [33]. The proposed method could be improved by incorporating machine learning methods in the outcome and propensity score modeling [34].

## Funding

Yu‐Han Chiu was supported by the National Institute of General Medical Sciences of the National Institutes of Health under award number R35GM154888. The content is solely the responsibility of the authors and does not necessarily represent the official views of the National Institutes of Health.

## Conflicts of Interest

The authors declare no conflicts of interest.

## Supporting information


**Data S1:** Supporting Information.

## Data Availability

The data that support the findings of this study are openly available in MissclassifiedT_latentVar at https://github.com/ys3298/MissclassifiedT_latentVar.
